# Global transcriptome analysis of rat hypothalamic arcuate nucleus demonstrates reversal of hypothalamic gliosis following surgically and diet induced weight loss

**DOI:** 10.1038/s41598-019-52257-8

**Published:** 2019-11-06

**Authors:** Pernille Barkholt, Kristoffer T. G. Rigbolt, Mechthilde Falkenhahn, Thomas Hübschle, Uwe Schwahn, Maria Luisa Fernandez-Cachon, Thorsten Schmidt, Stefan Theis, Henrik H. Hansen, Anders Hay-Schmidt, Philip J. Pedersen, Niels Vrang, Jacob Jelsing

**Affiliations:** 1 Gubra, Hørsholm, Denmark; 2 Department of Neuroscience and Pharmacology, University of Copenhagen, Copenhagen, Denmark; 3 Sanofi-Aventis Deutschland GmbH, Frankfurt Am Main, Germany

**Keywords:** RNA sequencing, Obesity, Metabolic disorders

## Abstract

The central mechanisms underlying the marked beneficial metabolic effects of bariatric surgery are unclear. Here, we characterized global gene expression in the hypothalamic arcuate nucleus (Arc) in diet-induced obese (DIO) rats following Roux-en-Y gastric bypass (RYGB). 60 days post-RYGB, the Arc was isolated by laser-capture microdissection and global gene expression was assessed by RNA sequencing. RYGB lowered body weight and adiposity as compared to sham-operated DIO rats. Discrete transcriptome changes were observed in the Arc following RYGB, including differential expression of genes associated with inflammation and neuropeptide signaling. RYGB reduced gene expression of glial cell markers, including *Gfap*, *Aif1* and *Timp1*, confirmed by a lower number of GFAP immunopositive astrocyte profiles in the Arc. Sham-operated weight-matched rats demonstrated a similar glial gene expression signature, suggesting that RYGB and dietary restriction have common effects on hypothalamic gliosis. Considering that RYGB surgery also led to increased orexigenic and decreased anorexigenic gene expression, this may signify increased hunger-associated signaling at the level of the Arc. Hence, induction of counterregulatory molecular mechanisms downstream from the Arc may play an important role in RYGB-induced weight loss.

## Introduction

Obesity and related co-morbidities are a major threat to both public health and health budgets across the world and the search for efficacious treatment options is becoming increasingly critical. Whereas lifestyle interventions and pharmaceutical treatments have had limited success in the treatment of obesity, bariatric surgeries are the only treatment options providing weight loss of a magnitude matching patient expectations^1,2^. Consequently, understanding the molecular mechanisms behind bariatric surgery could potentially lead to the development of new non-surgical approaches to the treatment of obesity.

The hypothalamus is known as the primary integrator of peripheral and central signals involved in homeostatic regulation of food intake and body weight^3^. In particular, the hypothalamic arcuate nucleus (Arc) is known to play a key role in sensing peripheral hormonal signals and relaying nutritional states to the brain, mainly ascribed to two distinct leptin and insulin responsive neuronal populations in the Arc. Dysregulated activity of arcuate neurons co-expressing the orexigenic neuropeptides neuropeptide Y (NPY) and agouti-related peptide (AgRP) and neurons co-expressing the anorexigenic peptides cocaine- and amphetamine-regulated transcript (CART) and pro-opiomelanocortin (POMC)^4–6^ is, at least in part, known to be responsible for caloric overconsumption and onset of obesity^7,8^. Recently, pro-inflammatory signaling mechanisms in the Arc have also been implicated in the pathogenesis of obesity^9,10^.

To date, a major focus in bariatric surgery research has been on the impact on gut-derived hormones, bile acids, microbiota and other peripheral adaptations^11–13^. In contrast, only a few studies have addressed central mechanisms involved in post-surgical satiety and weight loss. Accordingly, two independent studies have indicated transcriptional upregulation of arcuate orexigenic markers in rat models of biliopancreatic^14^ and duodenal-jejunal bypass^14,15^. Using quantitative *in situ* hybridization histochemistry (ISHH), we have recently reported similar findings in a rat model of Roux-en-Y gastric bypass (RYGB)^16^. As orexigenic melanin concentrating hormone (MCH) gene expression in the lateral hypothalamus was unaffected by RYGB, this suggested that compensatory overeating after RYGB surgery is prevented by recruitment of counterregulatory molecular signaling pathways downstream from the Arc^16^. The present study aimed to gain further insight into the gene regulations in the Arc following RYGB. To this end, laser-capture microdissection (LCM) and RNA sequencing were combined to determine global gene expression in the Arc after RYGB-induced weight loss in the diet-induced obese rat.

## Materials and Methods

### Animals

The Danish Animal Experiments Inspectorate approved all experiments which were conducted using internationally accepted principles for the use of laboratory animals under the personal license #2013-15-2934-00784. Male Sprague-Dawley rats (8 weeks old) were obtained from Taconic (Lille Skensved, Denmark). Upon arrival, rats were single-housed and maintained in controlled environmental conditions (12 h light/12 h dark cycle; 22 ± 1 °C; 50 ± 10% relative humidity). Rats were fed a two-choice diet consisting of chow (Altromin 1324, Brogaarden Denmark) and a high-palatable high-fat diet (HPHF diet; Nutella, peanut butter and powdered chow), as described previously^17^. Rats had *ad libitum* access to water and the two-choice diet regimen for 4 (4w DIO, n = 16) or 12 (12w DIO, n = 30) weeks to induce mild or severe obesity, respectively, prior to surgical intervention. For 12w DIO rats, body weight and food intake was monitored daily throughout the study. Body weight and food intake in the 4w DIO rats has been reported previously^16^.

### Surgical procedures

Rats were assigned to RYGB (4w DIO, n = 8; 12w DIO, n = 10) or sham surgery (4w DIO, n = 8; 12w DIO, n = 10). Sham-operated and weight-matched (WM) rats (n = 10) served as an additional control group to RYGB in the 12-week pre-feeding study. Three days prior to surgery, animals were placed on a liquid diet (Osmolite 1 Cal, Abbott Nutrition, Chicago, IL; Fresubin Original, Mediq Danmark, Broendby, Denmark). On the day of surgery, animals underwent whole-body composition analysis by non-invasive EchoMRI scanning (EchoMRI-900 Analyzer, EchoMRI, USA). The RYGB surgical procedure were carried out as previously described in detail^16^. In brief, the abdomen was exposed using a midline laparotomy after induction of surgical anesthesia with an isoflurane/O_2_ mixture. The jejunum was transected 30 cm distal to the ligament of Treitz, and a longitudinal anti-mesenteric incision was made 10 cm distal to the transected bowel and connected to the afferent limb of the jejunum with a running absorbable suture (Ethicon, Somerville, NJ, USA). The stomach was exposed, the fundus was excised and a staple line was placed across the waist of the stomach, creating a gastric pouch approximately 10% the size of the normal stomach. The distal remnant was returned to the peritoneal cavity and an incision was made on one side of the gastric pouch considering the vascular architecture. The efferent limb of the transected jejunum was connected to the gastric pouch with a running suture. After repositioning the gastric pouch into the peritoneal cavity, the abdominal wall was closed. The sham procedure followed the steps of the RYGB but gastrointestinal surgery only included a transection of the jejunum 30 cm distal to the ligament of Treitz, which was immediately re-sutured. Animals were subcutaneously administered with warm saline (37 °C, 20 ml/kg), enrofloxacin (0.5 mg/kg) and carprofen (0.5 mg/kg) to prevent post-operative infection, lethargy and pain relief. The wire grate was kept in place post-operatively for up to 10 days where only liquid diet was offered in this period whereupon the two-choice diet was reintroduced. The food ration for 12w DIO-WM rats was adjusted on a daily basis to promote a body weight change equal to that of 12w DIO-RYGB rats. A daily check was made to ensure that the previous food ration had been consumed.

### Termination and tissue sampling

60 days post-surgery, animals were euthanized by decapitation under CO_2_/O_2_ anesthesia. All animals were euthanized in the morning 2–3 hours after lights on. RYGB and sham-operated animals were euthanized in a randomized order. Prior to euthanasia, animals underwent an additional whole-body echo-MRI scan. Following decapitation, brains were quickly removed and snap-frozen on crushed dry ice and stored at −80 °C until further processing.

### Plasma sampling and hormone assays

Blood samples were collected at termination in EDTA-coated tubes containing DDP-IV inhibitor (DPP4-010, Millipore, Denmark) and protease inhibitor cocktail (P2714-1BTL, Sigma-Aldrich, Denmark). Plasma was separated and stored at −80 °C. Leptin, C-peptide, peptide YY (PYY) and ghrelin were determined in a rat multiplex assay (#RMHMAG-84K, Merck Millipore, Darmstadt, Germany) on a Luminex Flexmap 3D machine (Luminex, Austin, TX). Total and active GLP-1 was determined using single-plex assays (K150JVC/K150JWC, Meso Scale Discovery, Rockville, MD) on a MESO QuickPlex SQ 120 instrument (Meso Scale Discovery, Rockville, MD).

### Laser-capture microdissection and RNA sequencing

**Figure 1:**
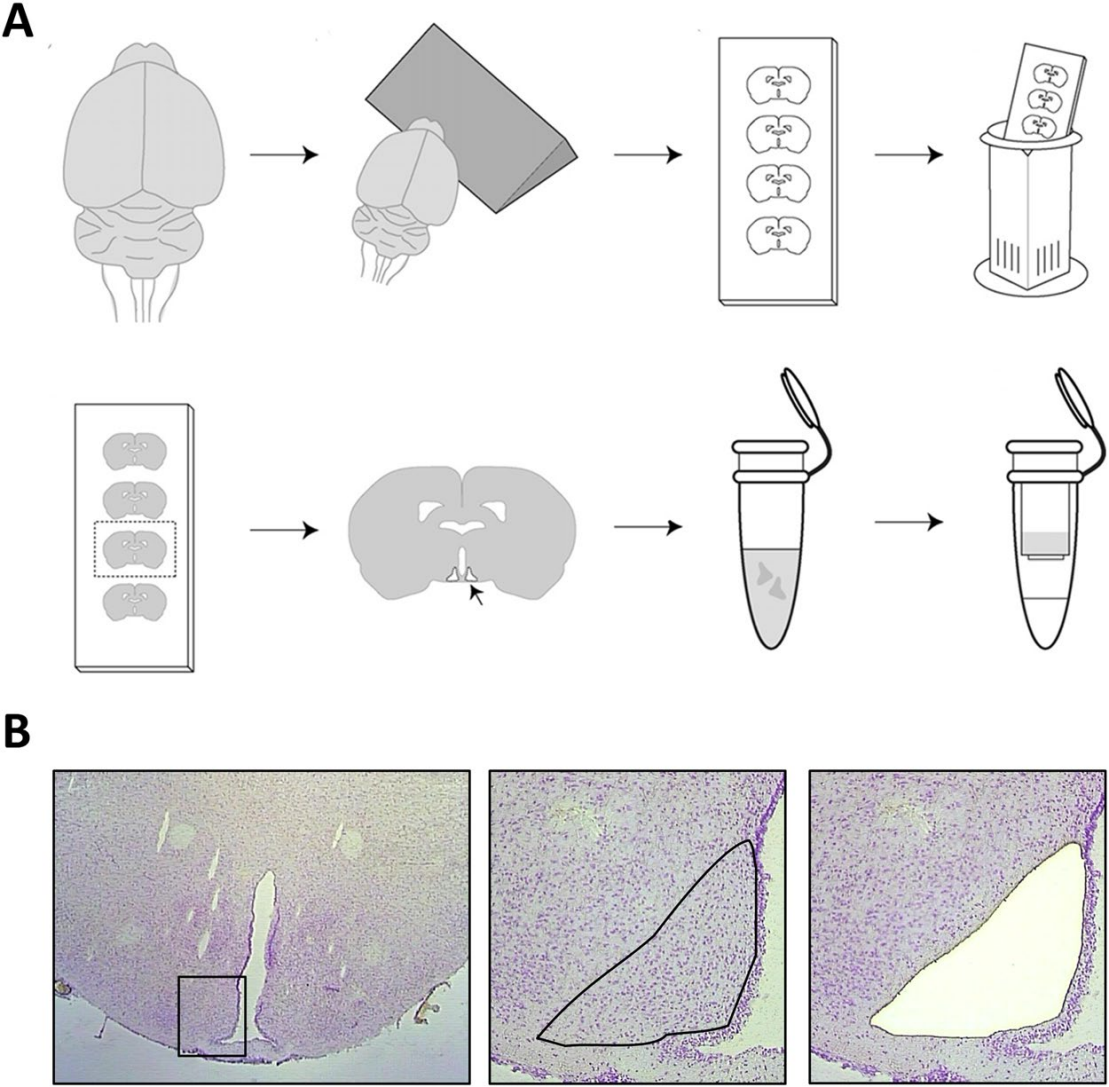
Laser-capture microdissection of the Arc. (**A**) Illustration of workflow from sectioning of brains collecting series of hypothalamic sections through cresyl violet staining to laser capture microdissection (LCM) of the Arc and RNA purification; adapted from Paulsen *et al*.^49^. (**B**) Hypothalamic section before and after LCM of the Arc.

The brains were sectioned on a cryostat (CM 3050, Leica, Germany) into 12 μm thick coronal sections using systematic uniform random sampling (Fig. 1A). Serial sections covering the entire rostro-caudal axis of the Arc were collected at every 180 μm (i.e. every 15^th^ section) on Pen membrane microscope slides (Life Technologies, Carlsbad, CA) for LCM and on super-frost plus slides for immunostaining, respectively. For LCM, sections were allowed to thaw for 5 min at 4 °C and then stained for 5 min in a 0.1% cresyl violet acetate (Sigma-Aldrich, St. Louis, MI) solution dissolved in 70% ethanol. Sections were then dehydrated in 96% and 100% ethanol at 4 °C and finally allowed to dry in fume hood for 2 min at room temperature. Using the Arcturus^XT^ LCM Instrument (AXT2148, Life Technologies, Carlsbad, CA) the Arc was identified and captured by a combination of infrared capturing and UV laser cutting. The Arc tissue was captured on CapSure® Macro LCM Caps (Applied Biosystems, Foster City, CA). The Arc was collected on 8–10 sections representing the entire rostro-caudal axis of the nucleus (Fig. 1B). Lysis of cells and RNA extraction was performed using the PicoPure RNA isolation kit (Life Technologies, Carlsbad, CA) as recommended. RNA integrity was assessed using the Bioanalyzer 2100 system (Agilent Technologies, Waldbronn, Germany). RIN (RNA integrity number) values ranged between 7.3–8.1 (data not shown). RNA amplification and sequencing was performed on all Arc samples by Cofactor Genomics (Saint Louis, MO). In brief, RNA sequencing (RNAseq) libraries were prepared with the Ovation® RNAseq system (NuGen, Redwood City, CA) and sequenced on the HiSeq platform (Illumina, San Diego, CA) (single-end, approximately 20 M 75 bp reads). Reads were aligned to the Rnor_5.0 Ensembl Rattus norvegicus genome using STAR v.2.3.0 and feature counts were obtained with HTseq v. 0.6.1, both with default parameters^18,19^. Known orexigenic/anorexigenic genes (AgRP, NPY, CART and POMC) were selected *a priori* for analysis of the orexigenic state in the Arc following RYGB. For network analysis a raw *p*-value < 0.01 was used as threshold and protein-protein interaction annotations were obtained from the String database^20^ and imported to Cytoscape^21^ for visualization. To identify putative associations of gene expression patterns, the Cytoscape plug-in BINGO^22^ was used to extract enriched GO terms for each network.

### Immunohistochemical analysis of astrocyte profiles

In all individual rats, an additional series of cryo-sections was used for glial fibrillary acidic protein (GFAP) immunohistochemistry. Sections were fixated in 10% neutral buffered formalin for 10 min followed by rinsing in water. Endogenous peroxidase activity was blocked for 10 min in 1% H_2_O_2_ + tris buffered saline (TBS) + 0.3% Triton-X (TX). Blocking of unspecific binding was obtained with 5% swine serum in TBS + 0.3% TX + 1% BSA for 20 min followed by addition of rabbit anti-GFAP antibody (1:300; Z0334; Dako, Denmark) diluted in TBS-T + 1% BSA for 60 min. The reaction was amplified for 30 min using MACH 2 rabbit HRP-polymer (RHRP520, BioCare Medical, USA). Sections were rinsed in TBS before subjected to 3,3′-diaminobenzidine as chromagen (K3467; Dako, Denmark). The evaluation of astrocyte profiles was performed on digitalized slides using the newCAST software (Visiopharm, Hørsholm, Denmark). A region of interest was delineated and a counting frame was placed using systematic uniform random sampling principles. Astrocyte profiles were counted when located within the counting frame. A total number of approximately 50 counting frames was evaluated.

### Statistics

Graphical presentations, calculations and statistical analyses were carried out using GraphPad software (GraphPad Prism version 5, San Diego, California, USA). Statistical analysis of *in-vivo* data, plasma analyses and mRNA levels of a priori selected genes were performed using either one-way analysis of variance (ANOVA) followed by Tukey’s *post-hoc* test or student’s unpaired t-test where appropriate. A *p* value less than 0.05 was considered statistically significant. Differential gene expression analysis was performed using edgeR with raw p-values^23^. Combined p-values were calculated using Fisher’s combined probability test applied separately to genes up-regulated or down-regulated in both cohorts.

## Results

### RYGB induces significant weight loss and increases plasma GLP-1 and PYY levels

Metabolic parameters are indicated in Table 1. We have previously reported significantly reduced terminal body weight and whole-body fat mass in 4w DIO-RYGB rats compared to 4w DIO-sham controls^16^. Correspondingly, 12w DIO-RYGB rats showed significant reductions in terminal body weight relative to baseline (F(2,27) = 4.52, p < 0.001). Also, 12w DIO-RYGB rats exhibited lowered terminal whole-body fat mass as compared to 12w DIO-sham controls (F(2,27) = 5.41, p < 0.05). The magnitude of absolute and relative weight loss as well as whole-body fat mass reductions in 12w DIO-RYGB rats were comparable to that of Sham-WM rats. Terminal lean mass was unaffected by RYGB in 12w DIO rats and slightly reduced in Sham-WM rats (F(2,27) = 3.67, p < 0.05). Cumulated energy intake was similar between RYGB and Sham rats in 12w DIO rats whereas Sham-WM rats were significantly food restricted (both chow and HPHF diet) in order to match the weight loss of RYGB rats (F(2,27) = 24.81, p < 0.001). Furthermore, 12w DIO-RYGB rats had an increased cumulated chow intake as compared to 12w DIO-sham controls (F(2–27) = 18.54, p < 0.001). Terminal plasma hormone levels in 12w DIO rats were significantly changed by RYGB (Table 1). Leptin levels were significantly reduced in 12w DIO-RYGB, but not Sham-WM rats, compared to 12w DIO-sham controls (F(2,27) = 6.08, p < 0.01). C-peptide levels were significantly reduced in both 12w DIO-RYGB and 12w DIO-WM rats (F(2,27) = 9.72, p < 0.001). Plasma total GLP-1 were significantly increased in 12w DIO-RYGB rats, as compared to both 12w DIO-sham and Sham-WM rats (F(2.27) = 16.18, p < 0.001). Active GLP-1 levels were significantly increased in 12w DIO-RYGB rats compared to both 12w DIO-sham controls (t = 5.37, df = 17, p < 0.01). While total GLP-1 levels were unchanged in 12w DIO-WM rats, active GLP-1 concentrations were below assay detection level (0.24 pg/ml) in 12w DIO-WM. Total PYY levels were significantly increased in 12w DIO-RYGB rats compared to both 12w DIO-sham and 12w DIO-WM rats (F(2.27) = 57.46, p < 0.001). Total PYY levels were unchanged in 12w DIO-WM rats. Plasma ghrelin levels were only increased in 12w DIO-WM rats (F(2.27) = 4.23, p < 0.05). These metabolic effects are in agreement with the consistent findings in clinical studies on RYGB^24–26^, supporting the translational value of the present DIO rat model of RYGB.

**Table 1:** *In vivo* endpoints and plasma hormone levels.

	DIO-Sham	DIO-RYGB	DIO-WM
4 weeks of HPHF feeding prior to surgery (4w DIO)			
Pre-surgical body weight (g)	360 ± 7	409 ± 16	—
Terminal body weight (g)	522 ± 19	442 ± 16**	—
Body weight change relative to day-1 (%)	153 ± 6	110 ± 4***	—
Cumulated chow intake (g)	160 ± 43	448 ± 76**	—
Cumulated HPHF diet intake (g)	912 ± 42	768 ± 69*	—
Cumulated energy intake (kJ)	23 777 ± 627	23 756 ± 1374	—
Pre-surgical fat mass (g)	35 ± 3	53 ± 6*	—
Terminal fat mass (g)	98 ± 10	45 ± 3***	—
Pre-surgical lean mass (g)	289 ± 5	341 ± 16*	—
Terminal lean mass (g)	371 ± 7	373 ± 17	—
**12 weeks of HPHF feeding prior to surgery (12w DIO)**			
Pre-surgical body weight (g)	728 ± 22	745 ± 23	762 ± 27
Terminal body weight (g)	725 ± 20	620 ± 15**	636 ± 21*
Body weight change relative to day-1 (%)	99.7 ± 1.1	83.4 ± 0.9***	83.5 ± 0.4***
Cumulated chow intake (g)	51 ± 10	267 ± 42***	163 ± 5*^/^^
Cumulated HPHF diet intake (g)	826 ± 26	861 ± 66	538 ± 16***^/^^^^
Cumulated energy intake (kJ)	20 420 ± 586	23 818 ± 1 506	14 856 ± 418***^/^^^^
Pre-surgical fat mass (g)	220 ± 23	224 ± 20	248 ± 13
Terminal fat mass (g)	222 ± 22	147 ± 18*	160 ± 9*
Pre-surgical lean mass (g)	475 ± 8	493 ± 9	467 ± 13
Terminal lean mass (g)	489 ± 12	475 ± 8	452 ± 11*
Leptin (pg/mL)	4218 ± 413	2716 ± 277**	3209 ± 204
C-peptide (pg/mL)	1269 ± 118	807 ± 51**	846 ± 61*
GLP-1 total (pg/mL)	8.2 ± 1.1	44.9 ± 7.8***	7.9 ± 4.4^^^^^
GLP-1 active (pg/mL)	0.61 ± 0.7	3.28 ± 0.5**	Below LOD
PYY (pg/mL)	21 ± 2	138 ± 16***	18 ± 1^^^^^
Ghrelin (pg/mL)	146 ± 27	145 ± 28	234 ± 15*^/^^

Roux-en-Y gastric bypass (RYGB) and weight-matching (WM) led to a significant reduction in plasma leptin levels compared to sham-operated controls. RYGB led to an increase in the plasma levels of gut-derived hormones GLP-1 (total and active) and PYY relative to both sham and Sham-WM, whereas only Sham-WM showed significantly increased plasma levels of ghrelin. LOD, level of detection (active GLP-1 was below detection level in sham-WM animals (0.24 pg/mL)). Cumulated energy intake includes chow and high palatable high fat (HPHF) diet consumption over the course of the experiments. Data on body weight and body composition in the 4w DIO rat study have previously been reported^16^. Data are presented as mean ± SEM; *p < 0.05 vs sham, **p < 0.01 vs sham, ***p < 0.001 vs sham; ^^^*p* < 0.05 vs RYGB, ^^^^^*p* < 0.001 vs RYGB (Student’s unpaired t-test or one-way ANOVA with Tukey post-hoc test).

### Global gene expression analysis in the arcuate nucleus

LCM on the Arc was used to determine a region-specific transcriptional profile of gene regulatory events following RYGB vs. sham-operated and sham-WM controls, respectively (Fig. 1). To increase confidence in the observed differential expression, arcuate RNAseq data sets from DIO-RYGB rats (4w DIO-RYGB, 12w DIO-RYGB) and DIO-sham rats (4w DIO-sham, 12w DIO-sham) were pooled and analyzed specifically for differentially expressed genes (DEGs) regulated in the same direction in both 4w and 12w DIO-RYGB rats. Using this procedure, a total of 21 DEGs were identified (combined FDR <0.05), including 13 downregulated and 8 upregulated genes, respectively (Table 2). Most significantly down-regulated genes were associated with astroglial (*Gfap*, *Aqp4*, *Fabp7*) and immune cell (*Serping1*, *Fabp4*) function. In addition, RYGB promoted significant downregulation of genes associated with extracellular matrix (ECM) remodeling and cell migration (*Timp1*, *Tmbim1*, *Capn3*, *Ucma*). Significantly upregulated genes represented transcription factors (*Egr1*, *Egr2*), nuclear receptors (*Nr4a1*, *NR4a3*) as well as the orexigenic peptide, agouti-related peptide (*Agrp*). The set of 21 genes were more robustly regulated in 4w DIO-RYGB rats compared to 12w DIO-RYGB rats (Table 2).

**Table 2:** Differentially expressed genes in the n. arcuatus of RYGB-operated DIO rats.

Ensembl ID	Gene	Function	4w DIO-RYGB				12w DIO-RYGB				Combined	
			RPKM	Ratio	Raw p-value	FDR	RPKM	Ratio	Raw p-value	FDR	Raw p-value	FDR
**Downregulated**												
ENSRNOG00000002919	Gfap	Astroglial cell morphology	429	0.38	1.37E-09	1.02E-05	428	0.73	5.08E-06	5.46E-02	2.34E-13	1.52E-09
ENSRNOG00000010208	Timp1	Extracellular matrix remodeling, cell migration	47	0.44	2.25E-07	1.11E-03	22	0.82	4.99E-03	8.00E-01	2.43E-08	6.71E-05
ENSRNOG00000010805	Fabp4	Fatty acid trafficking, inflammation	2.0	0.07	8.19E-11	1.22E-06	0.2	0.49	1.19E-01	9.95E-01	3.06E-07	1.94E-03
ENSRNOG00000016043	Aqp4	Astrocyte water homeostasis	300	0.67	2.57E-04	1.69E-01	905	0.85	7.91E-04	3.92E-01	3.33E-06	3.62E-03
ENSRNOG00000000814	Fabp7	Fatty acid trafficking, glial cell proliferation	296	0.56	3.36E-05	4.54E-02	81	0.88	8.89E-03	8.00E-01	4.80E-06	4.47E-03
ENSRNOG00000013654	Cbln2	Synapse assembly	28	0.66	2.58E-03	4.79E-01	33	0.80	1.59E-04	2.67E-01	6.44E-06	5.25E-03
ENSRNOG00000007457	Serping1	Regulation of complement cascade	13	0.57	3.73E-03	5.33E-01	15	0.78	1.87E-04	2.67E-01	1.06E-05	6.92E-03
ENSRNOG00000058906	Ptprh	Regulation of cell growth	0.6	0.25	2.50E-06	6.19E-03	2.0	0.92	5.54E-01	9.95E-01	2.01E-05	1.19E-02
ENSRNOG00000023546	Hspb1	Regulation of cell growth	8.3	0.46	5.81E-05	5.51E-02	4.8	0.82	2.75E-02	8.98E-01	2.29E-05	1.25E-02
ENSRNOG00000006735	Cdkn2b	Regulation of cell growth	1.3	0.38	1.99E-06	5.51E-02	3.4	0.84	1.88E-01	9.95E-01	5.90E-06	1.55E-02
ENSRNOG00000014797	Tmbim1	Extracellular matrix remodeling, cell migration	29	0.66	2.15E-05	3.78E-02	26	0.94	1.28E-01	9.63E-01	3.81E-05	1.78E-02
ENSRNOG00000008609	Capn3	Extracellular matrix remodeling, cell migration	3.0	0.49	1.29E-04	1.01E-01	6.7	0.85	3.17E-02	9.00E-01	5.50E-05	2.39E-02
ENSRNOG00000017987	Ucma	Extracellular matrix remodeling, cell migration	12	0.41	9.79E-06	5.51E-02	2.4	0.75	1.51E-01	9.95E-01	2.14E-05	3.74E-02
**Upregulated**												
ENSRNOG00000019422	Egr1	Transcription factor	17	4.28	4.45E-07	1.65E-03	35	1.48	3.25E-03	6.70E-01	3.09E-08	6.71E-05
ENSRNOG00000039001	Agrp	Appetite stimulation	185	1.53	5.68E-03	6.68E-01	55	1.93	1.84E-05	8.83E-02	1.78E-06	2.90E-03
ENSRNOG00000000640	Egr2	Transcription factor	0.7	3.17	2.29E-05	3.78E-02	3.1	1.59	8.26E-03	8.00E-01	3.11E-06	3.62E-03
ENSRNOG00000002793	Sstr2	Hormone secretion, regulation of cell growth	6.0	1.79	1.98E-06	5.89E-03	14	1.06	3.39E-01	9.95E-01	9.99E-06	6.92E-03
ENSRNOG00000007830	Apold1	Endothelial cell signaling	1.7	2.83	1.96E-05	3.78E-02	4.8	1.19	1.17E-01	9.59E-01	3.22E-05	1.62E-02
ENSRNOG00000005964	Nr4a3	Nuclear receptor	2.8	2.14	2.87E-05	4.27E-02	16.6	1.09	1.62E-01	9.70E-01	6.16E-05	2.51E-02
ENSRNOG00000007637	Acer2	Ceramide metabolism	4.8	1.45	2.16E-02	9.01E-01	27.5	1.28	3.51E-04	2.67E-01	9.72E-05	3.52E-02
ENSRNOG00000007607	Nr4a1	Nuclear receptor	2.7	3.07	2.67E-04	1.69E-01	5.4	1.33	4.09E-02	9.00E-01	1.36E-04	4.66E-02

Arcuate transcriptome analysis was performed 60 days following RYGB. Data are shown for individual experiments as well as following combined analysis. Combined statistical analysis of RNAseq data from both 4w DIO and 12w DIO rats indicated differential gene expression (FDR <0.05) of 21 genes (downregulated, n = 13; upregulated, n = 8) following RYGB, as compared to sham-operated controls. Genes are ranked according to combined FDR value. Gene expression level of individual genes is indicated by RPKM value. Ratio indicates gene expression level following RYGB surgery relative to corresponding sham group (RYGB/sham).

### Network analysis and histological validation

**Figure 2:**
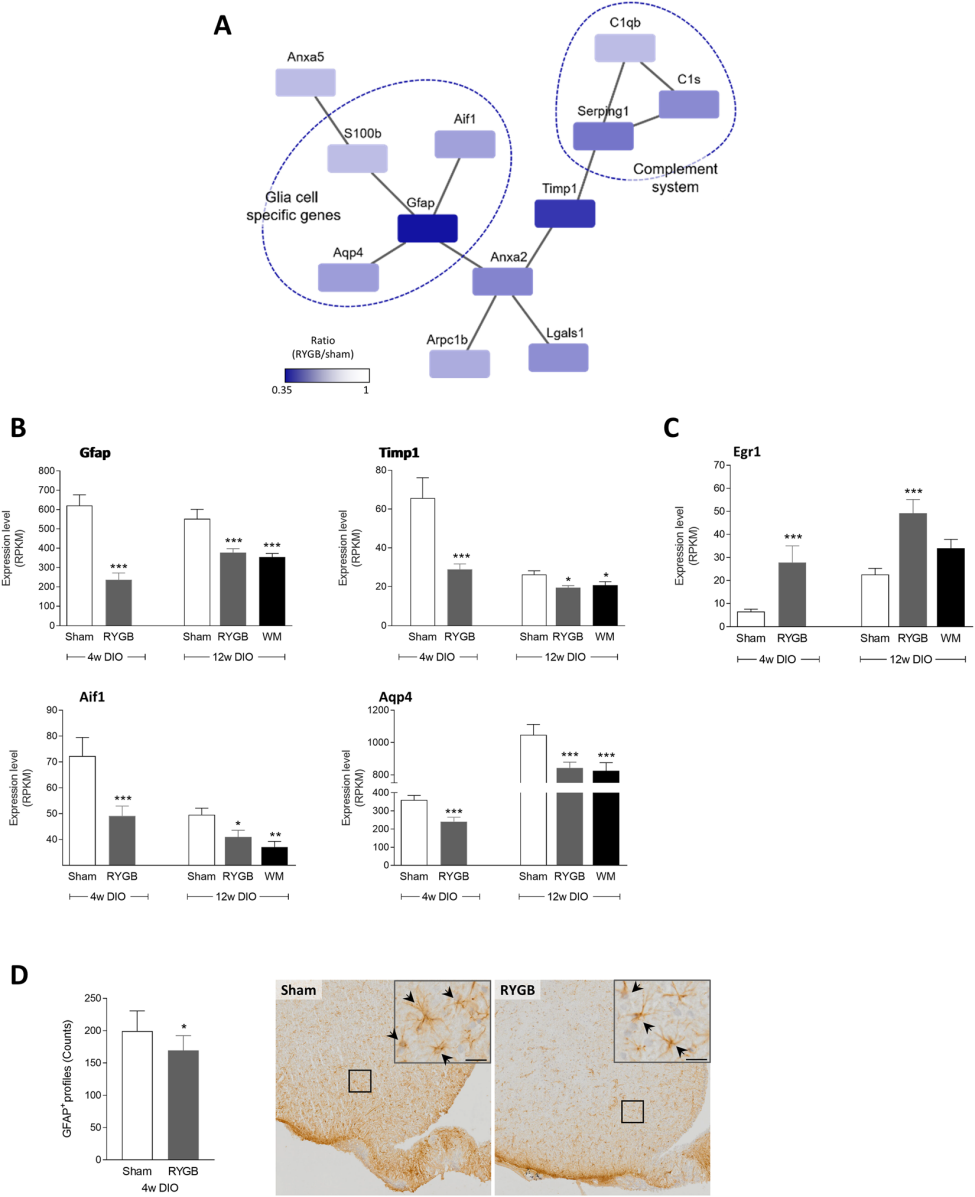
Reduced expression of inflammatory marker genes after RYGB. (**A**) Network analysis of all genes with raw *p*-values below 0.01 demonstrated several genes specific for glia cells as well as the complement immune system to be downregulated in RYGB-operated DIO (DIO-RYGB) rats relative to sham-operated DIO (DIO-sham) rats. (**B**) Expression levels of glia cell marker genes were downregulated following RYGB surgery in both studies (scale bar, 20 µm). (**C**) The transcription factor *Egr1* was upregulated in both 4w and 12w DIO-RYGB rats. (**D**) Expression levels are presented as mean reads per kilobase million (RPKM) ±SEM; statistical analysis was performed using EdgeR presented with raw *p*-values; *p < 0.05, **p < 0.01, ***p < 0.001. (**D**) The number of GFAP-positive astroglia profiles was significantly downregulated in 4w DIO-RYGB relative to 4w DIO-sham controls. Representative micrographs of GFAP-immunostained sections from sham-operated and RYGB-operated animals with arrowheads identifying astrocytes; scalebar equal to 20 µm in inserts. GFAP-positive cell counts are presented as mean ± SEM; *p < 0.05 (Student’s unpaired t-test). Gene names: *Aif1*, Allograft inflammatory factor 1; *Anxa2*, Annexin A2; *Anxa5*, Annexin A5; *Arpc1b*, Actin-related protein 2/3 complex subunit 1B; *Aqp4*, Aquaporin 4; *C1s*, Complement component 1s; *C1qb*, Complement component 1q B chain; *Egr1*, Early growth response protein 1 (also known as Zif268); *Gfap*, Glial fibrillary acidic protein; *S100B*, S100 calcium-binding protein B; *Lgals1*, Galectin-1 (lectin, galactose binding, soluble 1); *Serping1*, Serpin peptidase inhibitor, clade G (C1 inhibitor) member 1; *Timp1*, Tissue inhibitor of metalloprotease 1.

Subsequent network analysis of arcuate DEGs in 4w DIO-RYGB rats confirmed several significantly downregulated genes (raw p-value below 0.01) being associated with glial function, see Fig. 2A. Most marked reductions in expression levels (RPKM values) were observed for glia cell-specific genes, including *Gfap* and *Aqp4* (Fig. 2B). Also, the network analysis indicated lowered *Gfap* expression was linked to reduced expression of the microglial marker *Aif1* (also known as *Iba1*), see Fig. 2A,B. In addition, the network analysis confirmed reduced expression of genes functionally linking glial cell and complement function, as represented by significant downregulation of *Timp1* (Fig. 2A,B). 12w DIO-RYGB and 12w DIO-WM rats showed similar degree of downregulation of *Gfap*, *Aqp4*, *Aif1* and *Timp1* expression relative to 12w DIO-sham controls (Fig. 2B). Compared to 12w DIO-sham rats, *Egr1* expression was significantly down-regulated in 12w DIO-RYGB rats, but not 12w DIO-WM rats (Fig. 2C). To further confirm reduced expression of astroglial markers following RYGB, we performed quantitative immunohistochemical analysis of GFAP-positive astrocyte profiles in the Arc of 4w DIO rats. Compared to 4w DIO-sham rats, 4w DIO-RYGB rats showed significantly lowered numbers of GFAP-positive cell profiles (199 ± 11 vs. 169 ± 8, p < 0.05), see Fig. 2D.

### Analysis of a priori selected genes

**Figure 3:**
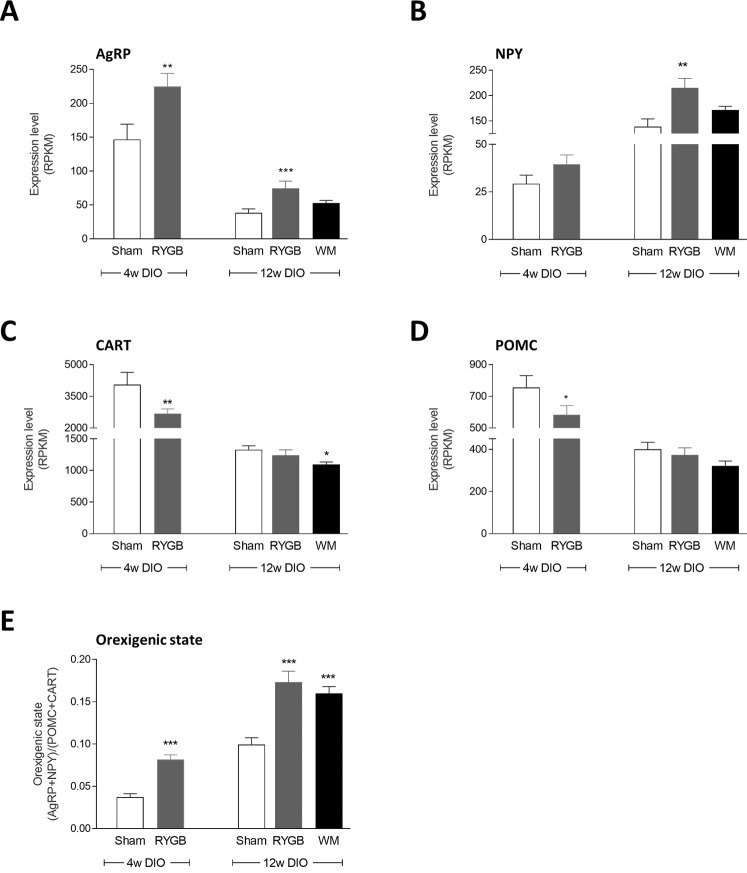
Differential expression of arcuate neuropeptide genes related to hunger and satiety signaling. Orexigenic AgRP (**A**) and NPY (**B**) mRNAs were elevated after RYGB in both studies relative to sham controls. Anorexigenic CART (**C**) and POMC (**D**) mRNAs were reduced in RYGB following 4 weeks, but not 12 weeks of pre-feeding. Statistical analysis was performed using EdgeR with raw *p*-values; *p < 0.05, **p < 0.01, ***p < 0.001 compared to corresponding sham control group. (**E**) The increased ratio between the expression level of orexigenic and anorexigenic genes suggests an orexigenic signaling state following RYGB compared to sham surgery; ***p < 0.01 compared with sham (student’s unpaired t-test or one-way ANOVA with Tukey post-hoc test). Data is presented as mean ± SEM.

We have previously reported potential changes in orexigenic signaling in 4w DIO-RYGB rats, as determined by ISHH analysis of arcuate AgRP/NPY and POMC/CART mRNA expressing neurons^16^. Combined LCM-RNAseq analysis of arcuate global gene expression confirmed significant upregulation of *Agrp* expression in 4w DIO-RYGB rats compared to 4w DIO-sham controls (p < 0.01). Similarly, *Agrp* expression was significantly upregulated in 12w DIO-RYGB rats (p < 0.001), but not 12w DIO-WM rats, see Fig. 3A. *Npy* expression was only significantly increased 12w DIO RYGB rats (p < 0.01, Fig. 3B). Both *Cart* and *Pomc* levels were significantly reduced in 4w DIO-RYGB rats (p < 0.01, p < 0.05). While *Cart* and *Pomc* levels were unchanged in 12w DIO-RYGB rats (Fig. 3C,D), 12w DIO-WM rats showed reduced expression of *Cart* (p < 0.05, Fig. 3C). The ratio of orexigenic (*Agrp* + *Npy*) vs. anorexigenic (*Pomc* + *Cart*) gene expression was calculated to provide an index of overall orexigenic signaling status of the Arc. This procedure revealed significantly increased ratios in both 4w DIO-RYGB (p < 0.001), 12w DIO-RYGB (p < 0.001) and 12w DIO-WM rats (all p < 0.001), as compared to corresponding sham controls, see Fig. 3E.

## Discussion

Here, we employed LCM in combination with RNAseq for a highly sensitive analysis of gene expression changes in the hypothalamic Arc following RYGB surgery in the DIO rat. Global gene sequencing indicated that the most marked transcriptional changes in the hypothalamic arcuate nucleus following RYGB were associated to markers of immune function. Notably, *Gfap* expression and number of GFAP-positive cell profiles were significantly downregulated compared to sham-surgery, suggesting reversal of reactive gliosis after RYGB-induced weight loss. Moreover, we extend previous findings based on quantitative ISHH technologies and confirm upregulation of arcuate orexigenic gene expression markers after RYGB surgery.

The initial identification of downregulated *Gfap* and *Timp1* expression suggested altered glia cell function upon RYGB surgery. Whereas *Gfap* is a well-known marker of astrocytes^27^, *Timp1* is known to be expressed by both neurons and astrocytes in response to insults such as ischemia, demyelination and kainate-induced seizures^28–30^. It has previously been demonstrated that high fat diet and obesity induce a state of inflammation in the hypothalamus, as indicated by an increased expression of proinflammatory marker genes (such as *IL-1β*, *IL-6*, *TNF-α*, and *Gfap*)^31,32^, supported by reports of increased number and ramifications of microglia^33^ and increased numbers of GFAP positive cells^34^. To further address potential inflammatory changes after RYGB, we performed a series of targeted bioinformatics analyses. A network analysis supported reduced expression of several glia cell marker genes, as well as genes related to the complement innate immune system, following RYGB. Astrocytes and microglia are dynamic cells that are important in maintaining the microenvironment surrounding neurons and in co-modulating neuronal functions, such as synaptic plasticity and metabolism^35,36^. In response to CNS insults, both cell types respond rapidly and develop a reactive phenotype termed reactive gliosis characterized by upregulation of specific structural proteins like GFAP in astrocytes^37^ and allograft inflammatory factor 1 (AIF-1) in microglia^38^. The reduction in both *Gfap*, *Aqp4* and *Aif1* expression after RYGB therefore suggests ongoing processes towards reversal of gliosis. Since data from WM controls demonstrated similar alterations, the reversing of gliosis appears to be an effect of weight loss *per se*, rather than specific to RYGB surgery. In line with this, Grayson and coworkers^39^ have reported reduced microglia infiltration in the hippocampus following RYGB, and pair-feeding leads to a similar improvement. RYGB also resulted in increased expression of the transcription factors *Egr1* and *Egr2*. Interestingly, this effect was not observed in WM animals. Although the implications of regulated *Egr1/Egr2* expression in the Arc is not fully understood, *Egr1* expression has been associated with appetite signaling. For example, central infusion of NPY increases Egr1 protein levels in the Arc, and downregulation of *Egr1* results in halted post-translational processing of POMC into α-MSH^40^. As systemic administration of ghrelin also upregulates arcuate Egr1 protein expression in both fasted and fed rats^41^, this suggests a complex role of Egr1 in both orexigenic and anorexigenic signaling.

Using quantitative ISHH, we have previously reported that RYGB increases NPY and AgRP mRNA expression in the Arc without affecting POMC and CART mRNA levels in 4w DIO-RYGB rats, suggesting increased orexigenic tone at the level of the mediobasal hypothalamus^16^. Using combined LCM-RNAseq, we confirmed upregulation of arcuate orexigenic signaling in both 4w and 12w DIO-RYGB rats. In addition, LCM-RNAseq analysis indicated reduced *Pomc* and *Cart* expression in 4w DIO-RYGB rats, but not 12w DIO-RYGB rats. The discrepancies may be related to differences in sensitivity using RNAseq compared to ISHH as well as differences in pre-surgical body weight. Nonetheless, taken together our data suggest enhanced arcuate orexigenic signaling in a DIO rat model of RYGB, and extend findings reported in other rat models of bariatric surgery, including biliopancreatic diversion^14^, vertical sleeve gastrectomy^42^ and gastric banding^43^. NPY/AgRP and POMC/CART expressing neurons are well-known metabolic sensors highly responsive to changes in feeding status and circulating levels of nutrients and hormones, including glucose, leptin and insulin (for review see^44^). As RYGB reduced plasma leptin and insulin levels, it may be speculated that improved metabolic status played a role in the adaptive changes in the Arc. RYGB also markedly increased plasma levels of GLP-1 and PYY levels, which are gut-derived anorectic hormones^45,46^. We have previously reported RYGB-induced hypertrophy of the rat gut mucosa in the food exposed regions of the small intestine, coupled with a doubling in the total number of L-cells and marked increases in intestinal preproglucagon and PYY mRNA expression^47^. Hence, the intestinotrophic effect of RYGB likely explains the markedly elevated plasma levels of GLP-1 and PYY in the DIO-RYGB rat. Because WM did not increase circulating levels of GLP-1 and PYY, this supports the notion that these hormones are not responsible for RYGB-induced weight loss, at least at the level of the Arc^48^.

## Conclusion

For the first time, we here present global gene expression analysis of the Arc in a rat model of RYGB surgery. By use of highly sensitive cutting-edge technologies (the combination of LCM and RNAseq), we demonstrate a marked modulation of immune signaling pathways following RYGB. In particular, mRNAs related to astrocyte and microglia function were reduced, suggesting lowered glia cell activity. These findings support that weight loss, whether induced by bariatric surgery or caloric restriction, may lead to improved regulation of immune function within the Arc.
